# Targeting tau in Alzheimer’s Disease: rationale, approach and challenges

**DOI:** 10.1186/s13024-026-00925-5

**Published:** 2026-02-05

**Authors:** Aram Aslanyan, Martha S. Foiani, Tatiana A. Giovannucci, Eric McDade, Karen E. Duff, Catherine J. Mummery, Michael Schöll, Kristin R. Wildsmith, Ross W. Paterson

**Affiliations:** 1 Dementia Research Centre, UCL Queen Square Institute of Neurology, University College London, London, UK; 2 National Hospital for Neurology and Neurosurgery, London, UK; 3 UK Dementia Research Institute at UCL, London, UK; 4 Department of Neurodegenerative Disease, UCL Institute of Neurology, Queen Square, London, UK; 5 UCL Great Ormond Street Institute of Child Health, London, UK; 6 Washington University School of Medicine in St Louis, St Louis, USA; 7 Institute of Neuroscience and Physiology, the Sahlgrenska Academy at the University of Gothenburg, Mölndal, Sweden; 8 Translational Science, Clinical Research, Eisai, Inc, Nutley, NJ USA; 9 Darent Valley Hospital, Dartford, UK

**Keywords:** Tau, Alzheimer’s Disease, Biomarkers, Therapeutics

## Abstract

Alzheimer’s Disease (AD) is the most common neurodegenerative disorder and the leading cause of dementia characterised by the accumulation of beta amyloid (Aβ) plaques and neurofibrillary tangles (NFT). Several monoclonal antibodies against amyloid in early AD have shown the utility of reducing brain amyloid in large phase 3 studies, resulting in modest clinical benefit. To further slow, or even halt disease progression, targeting additional pathobiological pathways is likely to be necessary. In this review, we aim to appraise the scientific rationale for targeting tau in AD. The burden of tau pathology is correlated with disease severity and its role in AD progression is complex, involving synapse dysfunction, neuronal loss, neuroinflammation and autophagy impairment. Evidence suggests that hypersecretion, post-translational modifications and aggregation propensity, dependent and independent of amyloid, are also linked to neurodegeneration. We review the therapeutic agents in development including tau synthesis modifiers, active and passive immunotherapies, post-translational modifiers and aggregation inhibitors. Finally, we consider the available biomarker tools for patient selection and drug effectiveness evaluation, and we identify key knowledge gaps that future novel biomarkers might address to make clinical trials of tau therapies more likely to succeed.

## Background

Brain accumulation of tau in the form of intracellular phosphorylated tangles is a pathological hallmark of Alzheimer’s Disease (AD). The cortical burden of tau pathology correlates with severity and progression of cognitive decline in AD [1–3] so targeting tau pathophysiology would be expected to ameliorate the clinical decline seen in AD. In this review, we appraise the scientific rationale for targeting tau. We consider the different therapeutic approaches and available biomarker tools, and what additional advances in biomarkers can support clinical development.

## Tau in human physiology

To understand the potential therapeutic effect of modulating tau, we start by considering the physiological role of tau – what is known about its function and whether we can safely modulate its biology.

Tau is encoded by the microtubule-associated protein tau (*MAPT)* gene on chromosome 17. *MAPT* is highly expressed in neurons [4] and alternative splicing yields 6 isoforms that differ by lacking or containing exons 2, 3 and 10. Isoforms containing exon 10 result in 4 microtubule binding region (MTBR) (4R) tau, and isoforms lacking exon 10 generate 3 MTBR (3R) [5]. Tau has multiple domains – N-terminus (1–165); proline-rich tau (166–242); MTBR tau (243–367) and C-terminus (368–441) [6]. We focus on 3R and 4R isoforms of tau as their function is well described in neurodegenerative disease and their alterations lead to different tauopathies and clinical phenotypes [7].

Intracellularly, tau promotes new microtubule formation by lowering the critical concentration of tubulin polymerisation [6, 8–12], therefore making it not only a stabiliser but also an inducer for microtubule formation [6]. Hence, tau supports the microtubules in adult neurons and suppresses microtubule shortening, which retains the structural integrity of axons required for sustaining several neuronal functions, such as mitochondria transport for localised energy production and synaptic health [8, 13, 14].

Tau knockdown or modification in humans has so far shown no serious safety concerns [15–20], supporting the view that therapies targeting tau would be well tolerated. By contrast, tau knockdown in cells and animal models has been associated with negative effects on mitochondria, nuclei, synapses, motor system, myelination, iron and glucose metabolism and tumour suppression and mild behavioural phenotypes [4, 21, 22]. However human and murine tau differ substantially in splicing (mice express mostly 4R tau, as opposed to a balanced expression of 3R and 4R isoforms in humans) and show great variability at the N-terminal sequence (11 amino acids in human tau are missing in mice) [23]. The significance of these differences is not fully understood.

## Tau pathophysiology in AD

Next, we examine the rationale for therapeutically targeting tau by considering its biological role in neurodegeneration.

### Tau hyperphosphorylation is a pathological hallmark of AD

Unmodified tau protein shows no intrinsic tendency for aggregation, however, post-translational modifications (PTM) change this characteristic [24]. Tau is a natively unfolded protein due to its low hydrophobicity and charge distribution [24]. It self-assembles into filamentous structures of aggregated and hyperphosphorylated tau with the help of kinases and phosphatases [25–30]. Hyperphosphorylation is thought to destabilize the association of tau with microtubules, leading to its relocation from axons to the soma and dendritic compartments [24, 31, 32].

It is still debatable whether phosphorylation or aggregation are the cause of tau toxicity. A recent paper, building on work from in vitro studies, shows that phosphorylation is sufficient to cause synapse loss and cognitive decline in a tau mouse model [33]. Furthermore, certain forms of tau phosphorylated at serine 231 and 217 are early indicators of pathogenic tau formation, and their levels are known to correlate well with disease progression [34, 35]. Since these soluble forms of tau may be more toxic than fibrils as seen in in vitro studies, these and other phosphorylation sites may be therapeutic targets as well as biomarkers of early disease that precede tangle formation [36].

Interestingly, tau hyperphosphorylation is not always pathological – for example, p-tau217 levels were shown to be significantly higher in healthy newborns compared to patients with AD and longitudinal analysis of preterm infants demonstrated gradual drop in p-tau217 levels over the first months of life, approaching the levels found in young adults [37]. Furthermore, phosphorylation is also known to occur in mammalian hibernation and is fully reversed upon arousal. The hibernation-related hyperphosphorylation involves some of the phosphosites affected in AD, however without MTBR tau aggregation [38]. Clearance of phosphorylated tau without any pathological sequelae indicates that there is a distinct regulatory mechanism that prevents tau aggregation.

### Other post-translational modifications of tau

Other PTM also contribute to misfolding and aggregation of tau. These include acetylation which has been shown to preclude tau degradation and contribute to pathology [39]. Ubiquitination targets the protein for degradation, which could overwhelm the system and promote tau aggregation in a negative feedback loop [40, 41]. Some of these markers such as Ub-K311, Ub-K317, Ub-K267 were found to be AD-specific [42–45]. The same study showed these ubiquitinated isoforms were able to discriminate between 4R and 3R tauopathies. This shows that these AD-specific ubiquitination sites may be useful for developing AD-specific assays in fluid samples, akin to the current use of phosphorylation sites.

Exploring the potential specificity/sensitivity of other PTM might be a fruitful avenue of research. For example, the 4R isoform is known to be predisposed to aggregation with initial phosphorylation, but cleavage of the C-terminus enables further fibrilization followed by increased phosphorylation in the proline rich region with acetylation and ubiquitination in MTBR [46, 47]. This demonstrates that post-translational markers can inform diagnostic as well as therapeutic criteria for choosing the correct therapeutic target depending on the stage of AD pathophysiology.

Another PTM is glycosylation. O-GlcNAcylation promotes microtubule binding and prevents phosphorylation and aggregation; conversely N-glycosylation, which is increased in AD, is found to promote impaired microtubule binding, tau phosphorylation and misfolding [48–50].

Finally, cleavage of tau by several proteases adds complexity by producing neo-epitopes that could be targeted with antibody-based therapies. Caspases such as Caspase-2 cleave tau at specific residues creating truncated species that are prone to further modifications, neurotoxicity and memory impairment [51]. Calpains, which are activated due to calcium dysregulation, cleave tau at non-caspase sites producing small fragments which are also neurotoxic [52, 53]. Several other proteases such as HtrA1 [54] and Cathepsins [55] are also implicated in tau breakdown with unclear relevance [51].

Taken together these data suggest that tau PTM, beyond phosphorylation, play important roles in AD progression, and relevant biomarkers are likely to play an important role in stratifying disease stage and selecting the correct therapeutic target [46].

### Tau aggregation and deposition

Tau aggregation plays an important role in AD pathogenesis. Tau forms small soluble aggregates, paired helical filaments (PHFs), straight filaments and twisted ribbons [48, 56]. The MTBR component of tau is a major constituent of insoluble tau aggregates in the brain and it is an important region for seeding and spreading of pathology [47, 57–62]. Tau misfolding and aggregation is propagated by tau seeding via a prion-like mechanism which can recruit unfolded monomeric tau into aggregates [63–65]. This prion-like behaviour was supported by an experiment in mice where tau fibril inoculation in non-transgenic mice induced tau inclusion in anatomically connected regions [66]. In a similar study in macaques AD-derived tau seeds were injected into the entorhinal cortex and they spread to hippocampus and cingulate cortex following Braak stages [67]. Furthermore, seed competent tau which precedes tau pathology along the Braak stages is enriched in synapses which highlights the mechanism of disease progression [68]. Therefore, identifying and targeting the seed competent PTM would be therapeutically important in the future [46].

### Tau is implicated in synapse dysfunction and loss

The goal of disease modification in AD is to slow down or stop the progression of cognitive impairment. Synaptic degeneration is the best neuropathological correlate of cognitive decline in AD and therefore it is theoretically a promising therapeutic target [69–71].

There is evidence that tau oligomers are specifically toxic to synapses preceding cell death [72]. Tau is also known to play an important role in synaptic functions such as long-term depression and long-term potentiation [73, 74]. Furthermore, studies in mice show that effects of tau in synapses can be reversed by reducing the levels of the protein, making a strong rationale to target tau to protect synaptic function in AD [73, 75–78].

The mechanism of tau interaction with synapses is not completely clear but it seems that tau can promote calcium influx via synaptic interaction which is toxic for synapses. This is different from beta amyloid (Aβ) synaptotoxicity which is thought to involve interaction with cell membrane receptors [69, 79–81]. Recent post-mortem studies in humans also showed that oligomeric tau was present in pre and post synaptic terminals even in areas with little fibrillar tau deposition suggesting that oligomeric tau accumulation in synapses occurs relatively early in AD [82].

### Role of tau in neuronal dysfunction and loss

Severe neuronal loss is a characteristic feature of AD in which tau is thought to play an important role [78]. Necroptosis is a biological pathway resulting in neuronal death in AD. It is executed by mixed lineage kinase domain-like (MLKL) protein, which is triggered by receptor-interacting protein kinase 1 and 3 (RIPK1 and RIPK3). Necroptosis positively correlates with Braak staging and is inversely correlated with brain weight and cognitive scores making it an important neurodegeneration biomarker [83, 84]. Phosphorylated tau (p-tau) stimulates neuronal death and intriguingly, necroptosis inhibitors (targeting RIPK1) negatively affect this process [85]. Furthermore, necroptosis inhibitor (targeting MLKL) reduced the load of p-tau in the hippocampus and improved spatial learning and memory deficits in rats, showing a link between tau and neuronal death [86, 87].

A more controversial perspective is that tangles and other insoluble forms of tau are not that toxic and do not cause critical neuronal loss; it is argued that soluble forms of tau are neurotoxic instead [88]. This is supported by pre-clinical models showing that tangles do not directly cause neuronal loss and cognitive decline [89, 90]. In contrast, oligomeric tau which traditionally was not detectable by classic stains, was found to be most propagated in the clinically fastest progressing phenotype [88, 91–93] suggesting that oligomeric tau species drive neurodegeneration, and they were just previously not seen by traditional staining methods.

Opinions on which forms of tau are toxic are divided, however it is crucial to further identify them as targeting the neurotoxic forms of tau will increase the likelihood of future therapeutic success.

### Tau induces neuroinflammation

Neuroinflammatory response is an acute and essential part of central nervous system protection and likely plays an important role in AD [94, 95].

Experimental models support a direct role of disease-associated microglia in tau pathology propagation [96]. It is hypothesized that there are two microglial activation peaks. The first peak may reflect the protective response of microglia in pre-clinical stages of AD and the pro-inflammatory response contributing to AD is the second peak [97–100]. While the initial neuroinflammatory response may alleviate the tau pathology in early stages (by activating microglia promoting the clearance of tau), sustained and increased inflammatory response to tau pathology exacerbates the pathology by overwhelming the phagocytic capacity and therefore promoting tau accumulation [101–103].

Microglial activation in response to tau is associated with pro-inflammatory cytokines (IL-1B, IL-6, TNF-alpha) [104–106] and they form a vicious cycle of tau pathology triggering further inflammation which in turn exacerbates tau pathology via direct pathways (by degrading pathological tau which promotes its accumulation) [107, 108] and indirect pathways (by activating tau kinases that increase tau hyperphosphorylation and aggregation) [105–107]. Several neuron-microglia signalling pathways have already been implicated in modulating tau pathology. CX3CR1 which is expressed in microglia and is responsible for regulating the efficiency of extracellular tau internalization and clearance, can cause excessive tau spread with genetic disruption [106, 107]. Likewise, TREM2 (which forms the microglial response to amyloid and tau pathology) knockdown facilitates seeding and spreading of tau aggregates [109].

Furthermore, microglia may be the missing intermediary component in Aβ-tau synergy and therefore an important part of therapeutic pathways. Soluble Aβ activates microglia, and the activated microglia act as vehicles for trans-synaptic tau transfer. They internalise the pathological tau, pack it into extracellular vesicles (EV) and release it, which can then be taken up by a neighbouring ‘healthy’ neuron [110–113]. This is a pathway that might contribute to tau spread in AD but not in other tauopathies.

### Tau overwhelms intracellular proteostasis mechanisms

The ubiquitin-proteasome system (UPS) and the autophagy-lysosome system are two major protein quality control mechanisms [114–117]. Pre-clinical data suggests that tau proteostasis is controlled by these two mechanisms. Likewise, tau pathology may have a negative effect on these systems.

The first proposed pathway involves the impairment of the UPS leading to accumulation of ubiquitinated proteins including tau as an early response to neuronal damage, and once cells are unable to clear the accumulated proteins, apoptosis starts and triggers caspase activation which leads to tau cleavage and overall blockage of protein degradation promoting tau pathology spread [118, 119].

The second pathway involves chaperone-mediated autophagy (CMA) which is responsible for clearance of multiple intracellular proteins with a specific motif including tau [114, 115, 119–121]. Acetylated tau, which could contribute to neurodegeneration by driving tau polymerisation into neurofibrillary tangles (NFT) [122–124], is cleared by macroautophagy (a type of non-selective autophagy) but it has been shown to inhibit CMA, which consequently decreases tau degradation. Accumulation of tau upon inhibition of CMA favours rerouting of oligomers of acetylated tau towards extracellular release, resulting in cell-to-cell propagation [114]. Furthermore, p62 which is a cargo protein that binds to autophagy proteins, co-localises with tau in AD. Accumulation of p62 is therefore a sign that various proteins that were destined to be cleared, were insufficiently degraded [125–128]. Therefore, stimulating this pathway could be another therapeutic target in the future.

Once the above systems fail, tau accumulates and inhibits these systems even further [114–117]. EV release can help to reduce intracellular tau accumulation, and they have been shown to slow down the disease by facilitating clearance of intracellular proteins into the extracellular space. However, the release of tau-containing EV can expedite the disease process by facilitating the dissemination of pathological proteins, triggering inflammatory response, angiogenesis and cell death [129].

### Tau hypersecretion is observed in the presence of amyloid plaques

The appearance of newly translated tau in human cerebrospinal fluid (CSF) was shown to be increased in the presence of amyloid plaques [130]. In stable isotope labelling studies in individuals with and without amyloid pathology, tau translation did not correlate with the burden of brain tau pathology measured using Positron Emission Tomography (PET), but increase in secretion was observed in the presence of amyloid plaques. The reasons for this hypersecretory state are unknown, but it may represent a compensatory mechanism to overcome the structural connectivity disruption caused by amyloid [130].

## Current approaches for targeting tau

**Fig. 1:**
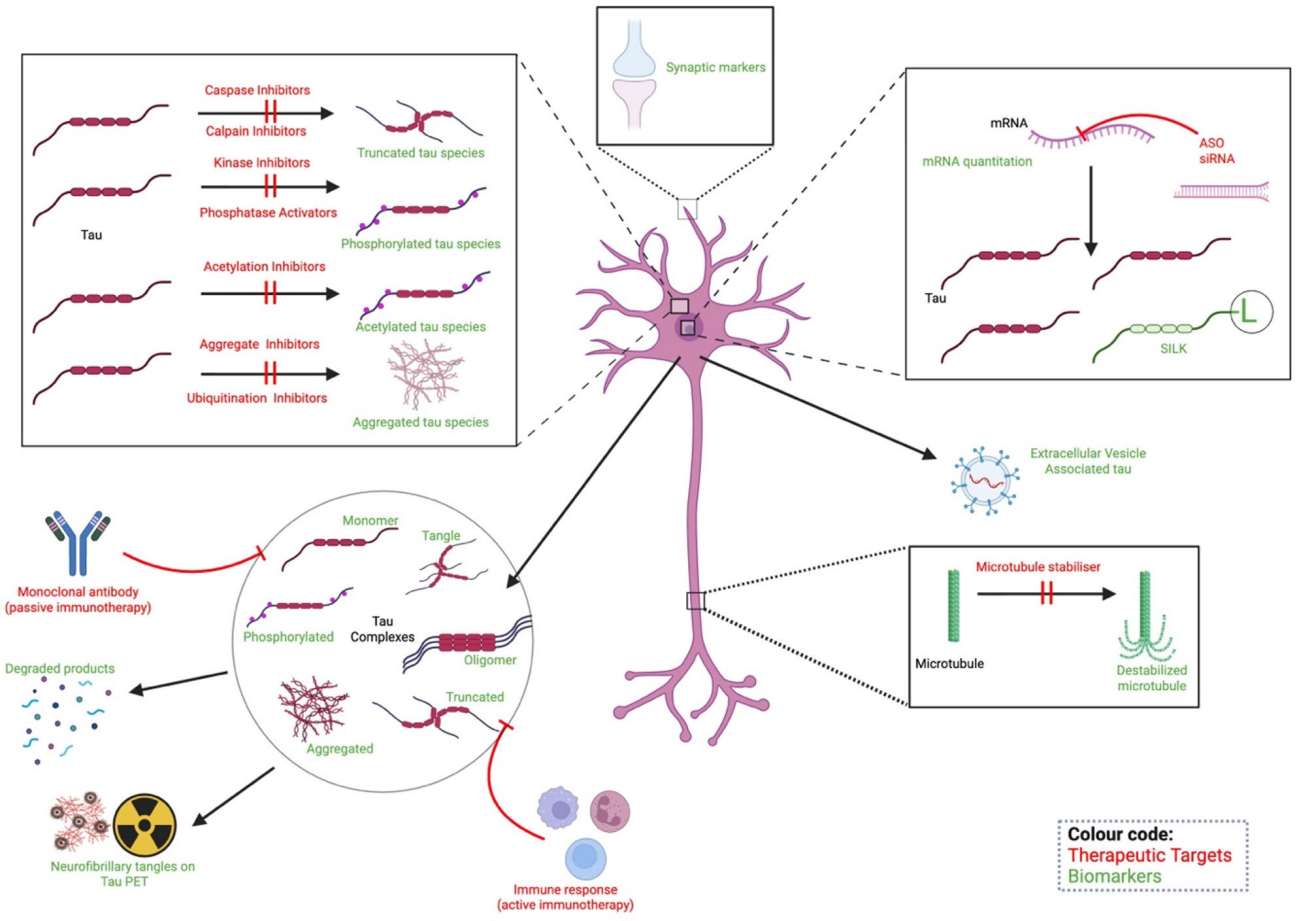
Current and hypothetical tau therapeutic targets and biomarkers. Schematic overview of tau-targeting therapeutic strategies (red) alongside potential biomarkers (green) for each mechanism (created with Biorender.com). Abbreviations: ASO, antisense oligonucleotide; mRNA, messenger RNA; SILK, Stable Isotope Labelling Kinetics; siRNA, small interfering RNA

The earliest AD therapeutics were traditionally focused on amyloid plaques in the 1980s and 1990s due to the prominence of the amyloid cascade hypothesis and relative ease of targeting extracellular amyloid pathology compared to other targets such as intracellular tau [131, 132]. The end of 1990s saw the first therapeutic targets focused on tau pathology and specifically tau hyperphosphorylation and aggregation [133]. Since then, there has been an exponential increase in AD clinical trials focusing on various targets related to tau. Various therapeutic modalities such as novel gene silencing and immunotherapies targeting immune cells have only been tried in cell/animal models [134, 135] which is beyond the scope of our review. We will be discussing tau therapeutic targets which have already made the translational move to human trials (Fig. 1).

There are 15 agents targeting tau in various phases in 2025 [136]. These trials have different targets which include targeting gene expression and reduction in production rate; antibody-mediated tau clearance; targeting post-translational pathological modifications or microtubule stabilisation and preventing tau seeding or aggregation [137].

## Modifiers of tau synthesis

A promising approach to addressing AD tauopathy is to address tau production by interfering with tau translation, effectively ‘turning down the tap’ and stopping possible overproduction as well as reducing the supply of tau into the aggregation process. There are two major approaches in achieving this.

### Messenger RNA (mRNA) therapy

Antisense oligonucleotides (ASO) are single-stranded oligodeoxynucleotides that can alter RNA and reduce or modify protein production via various mechanisms [138]. ASO therapy became even more popular in the field of neurodegeneration after it demonstrated target engagement and clinical benefit in Spinal Muscular Atrophy [139].

Animal studies have shown that ASO therapy targeting overexpression of human tau gene result in multiple positive outcomes such as reduction of p-tau and tau seeding activity, attenuation of brain volume loss compared to controls, and overall survival. Furthermore, social behaviour, cognitive and motor abilities were also improved. In non-human primates a reduction in mRNA and protein in the brain, spinal cord and CSF is observed [140, 141]. However, one concern with mRNA therapy that targets tau production is that it targets all forms of tau indiscriminately and reduced physiological tau could negatively impact normal physiology. Therefore, selectively modulating specific pathogenic isoforms, but preserving overall *MAPT* (tau) expression, may be a safer therapeutic approach [142]. Otherwise, a balance would need to be struck to reduce the pathological isoforms of tau sufficiently to cause the desired therapeutic effect without inhibiting the production of physiological tau excessively. The use of Stable Isotope Labelling Kinetics (SILK) has helped quantify the amount and timing of tau knockdown achieved in rats that received tau ASO therapy, by labelling new tau translation. This showed lowering of newly produced human tau earlier than levels of total tau, which suggested that the desired therapeutic effect (i.e. lowering of tau production) is achieved much earlier than anticipated [130, 143, 144].

Biogen’s tau ASO BIIB080, a *MAPT-*targeting ASO, is now in a phase 2 clinical trial in AD with no serious side effects seen so far, expected to finish the placebo-controlled phase in May 2026 [18, 19, 145]. BIIB080 reduced CSF tau concentrations in a dose dependent manner confirming target engagement, and early cognitive data suggested possible cognitive benefit [18, 19]. Furthermore, tau PET showed NFT reduction across all brain regions [18]; further supported by reduction in CSF MTBR-tau243 [146]. Novartis’ NIO752 is another *MAPT* ASO therapy, for which the phase 2 trial is scheduled to complete in 2025. NIO752 is also being investigated in a phase 1b study aiming to measure its effect on tau synthesis using SILK [20]. This study will help to quantify the amount of tau knockdown achieved with a *MAPT* ASO in real time and provide valuable information about how intracellular tau knockdown relates to more accessible peripheral and CSF tau biomarkers. This sort of novel approach may have important implications for dose selection in later phase studies.

### Tau small interfering RNA (siRNA)

An alternative strategy for interfering with tau translation is siRNA. These have shown considerable promise in reducing amyloid precursor protein (APP) as a strategy for addressing amyloid production in AD and a similar approach is likely to be possible for tau [15]. SiRNA has a long duration of effect due to catalytic recycling of the RNA-induced silencing complex (RISC). This may be advantageous for low frequency of intrathecal (IT) dosing. The effects of sustained and profound tau knockdown are not yet known, and safety signals may emerge late owing to the long half-life of tau in humans, making it particularly important to quantify the effects on intracellular tau translation.

In animal models, siRNA treatment durably reduced *MAPT* mRNA and protein in both in vitro and in vivo model systems. In non-human primate studies, there was an observed reduction of *MAPT* transcription and tau protein in brain tissue and CSF [135].

## Modifiers of tau spread

### Tau immunotherapy

Immunotherapy can be active or passive. Active immunotherapy delivers tau immunogen as a vaccine. Its advantages include low cost and long-lasting effect. However, since tau is an endogenous protein, there is the potential for an irreversible autoimmune response [48].

Passive immunotherapy delivers premade antibodies that target specific tau epitopes. The main advantage of passive immunotherapy is its flexibility - antibody subclasses can be easily changed, and they can be tailored to the disease stage. They are also relatively reversible. Disadvantages are high cost and the need for long-term administration [48].

### Active immunotherapy: Tau vaccination

This strategy is based on stimulating the immune system to produce antibodies that target tau. Tau vaccines are easily administered and can have long-lasting effects, targeting mainly the mid-region, MTBR and C-terminus [147]. There are various candidate antibodies [147].

In animals, vaccines that specifically mimic various phospho-epitopes of tau protein (out of 45 identified in AD brain) [148] have been found to be safe in mice and they led to reduced tauopathy in the brain [149, 150]. However, targeting a limited number of phosphorylated domains results in insufficient coverage and therefore limited therapeutic effect. Furthermore, some epitopes are also known to have a physiological function [151, 152] therefore the most effective vaccination options should avoid physiological epitopes and target pathological ones aiming to prevent tau prion-like seeding as well as tau aggregation and tangle formation [151]. Vaccinations are likely to be most beneficial in patients in pre-clinical stages of AD as immune responses are more likely to help stop the spread of tau pathology that is not yet extensive. This potentially makes them tools for primary prevention.

A concern with any vaccination is the autoimmune response which may be irreversible. Animal-based studies followed by early phase human studies have shown a benign safety profile to date [153]. Consideration also needs to be given to the variable immune response of the host immune system, leading to heterogenous antibody responses across patients; this may lead to difficulties when choosing the dose and vaccination regimen [147, 154, 155].

Currently two most advanced tau vaccinations in AD are AADvac1 and JNJ-64042056.

AADvac1 actively targets N-terminally truncated tau fragments and was shown to be safe in animal models [148]. AADvac1 showed no major safety concerns in participants with AD in phase 1 and showed lower hippocampal atrophy and better cognitive performance on participants with higher IgG responses [156, 157]. A phase 2 study also showed no major safety concerns (injection site reactions and transient confusion being main adverse events) but no significant changes were found in cognitive or functional tests [17]. It was later found that one-third of participants in this study were negative for tau biomarkers and post-hoc analysis of the subgroup with positive biomarkers showed that higher antibody titres correlated with clinical response and reduction in brain atrophy rate [158, 159]. The company is planning a larger phase 2b study of the vaccine [159].

JNJ-64042056 is a liposome-based vaccine designed to target pathological conformers of p-tau [160]. Animal models showed reduction in both soluble and insoluble tau as well as improved clinical parameters such as body weight retention, delayed motor phenotypes and extended lifespan with significant inflammatory response [149, 161]. In human trials the original vaccine showed a weak immune response which led to a vaccine redesign. Early phase studies showed good safety profile and titres for p-tau antibodies sustained for over a year. The company announced its phase 2/3 registration called Retain which will enrol participants with pre-clinical AD. The outcomes will be assessed by decline on the Pre-clinical AD Cognitive Composite 5 (PACC-5) with tau PET scans as secondary endpoint [161, 162].

### Passive immunotherapy: monoclonal antibodies

Tau is thought to spread from cell to cell through the extracellular space and propagate pathological forms in a prion-like fashion [163, 164]. Monoclonal antibodies are designed to block pathological extracellular tau spread from cell to cell and enhance tau microglial clearance thereby interrupting AD progression [163]. Many monoclonal antibodies, targeting a range of epitopes from the N-terminus through to C-terminus of tau, have been developed over the past decade. As a class they have shown a favourable side effect profile [16, 165–167]. There were no signs of Amyloid-Related Imaging Abnormalities (ARIA) which is an important advantage compared with amyloid-targeting monoclonal antibodies. However most (especially those targeting N-terminus [168] have not survived phase 1/2 clinical trials, due to lack of efficacy/target engagement, indicating that targeting the correct epitope and toxic species of the protein is likely to be important to prevent tau misfolding and spread [169–171].

Two promising tau antibodies target different epitopes within the R1-4 region on tau. One of the most promising tau monoclonal antibodies is Etalanetug, developed by Eisai, which is a monoclonal IgG1 antibody targeting HVPGG epitope in the MTBR [16]. Pre-clinical data showed reduced tau aggregation [16, 60]. A first in human phase 1 study showed adequate safety profile and target engagement [172] and in a recent phase 1b/2 study to assess safety and tolerability in Dominantly Inherited Alzheimer’s Disease (DIAD), a 30–70% decline in CSF MTBR-tau243 levels was observed albeit in a small sample size [57, 173, 174]. A phase 2/3 is running currently in patients with DIAD with the primary endpoint of tau spread measured by tau PET and other secondary outcomes such as the cognitive composite [16, 174, 175].

Similarly, bepranemab which is a full-length IgG4 monoclonal antibody developed by UCB Biopharma, showed a good side effect profile in its phase 1 trial [166]. Although it failed its primary outcome in phase 2, subgroup analysis of individuals with low baseline tau levels and no APOE4 (and therefore earlier tau pathology), showed that bepranemab slowed the cognitive decline by 50% on the Alzheimer’s Disease Assessment Scale-Cognitive Subscale (ADAS-Cog) compared to placebo. Importantly, this was the first drug to show slowing of tangle accumulation by 58% measured by PET ([^18^F]-GTP1). These subgroup analysis results warrant further investigation. The study also shows the importance of an individualised therapeutic approach taking into account factors such as APOE status [168].

## Tau accumulation modifiers

### Phosphatase modification

Tau hyperphosphorylation partially results from reduced protein phosphatase 2 A (PP2A) activity. Sodium selenate has been tested as a potential disease modifying therapy in AD as it reduces the hyperphosphorylated tau via activation of PP2A enzyme in mice models [176–181]. A study in humans showed a good safety profile and slowing of disease progression but it was not placebo-controlled [178] and phase 2 study showed only modest benefits on diffusion MRI [179].

### Kinase inhibition

Lithium chloride has been shown to inhibit glycogen synthase kinase 3B (GSK3B) which is known to phosphorylate tau [182]. In the first human studies there were no safety concerns [183], however phase 2 trial produced no clinical benefit, but the non-linear dose response suggested further dose finding studies and longer follow-up duration [184].

### Acetylation inhibition

Acetylation can impair microtubule binding, decrease solubility, promote cleavage and protein degradation impairment [48, 49, 114]. In this category salsalate has been shown to inhibit tau acetylation [185]. In mouse models, salsalate reduced total tau and acetylated tau levels, prevented hippocampal atrophy and reduced decline in memory deficits [124]. Phase 1 study in humans showed positive safety profile in patients with Progressive Supranuclear Palsy (PSP) [186], however the phase 1b trial in patients with AD is currently registered as active but not recruiting [187, 188].

### Caspase inhibitors

As mentioned, caspase is known to promote tau cleavage. Certain inhibitors such as minocycline and VX-765 were promising in animal models and showed reduced levels of tau phosphorylation and insoluble tau aggregates [189–191], however minocycline was trialled in humans with mild AD and failed to slow cognitive decline with higher doses associated with adverse effects [192].

### Microtubule stabilizers

TPI-287 which is used in oncology trials [193] was associated with reduced cognitive decline compared to placebo in AD in humans, however this was attributed to the above expected cognitive decline in the placebo group [194]. Further studies are needed to clarify its potential therapeutic benefits in AD.

### Tau aggregation prevention

The rationale for targeting O-GlycNAcase (OGA) comes from previous studies showing that addition of O-linked N-acetylglucosamine (N-GlcNAc) to tau reduces its propensity to form aggregates [195, 196]. The therapeutic strategy is therefore to use inhibitors of OGA – the enzyme that removes N-GlcNAc from proteins [196, 197]. Attractively, these therapies can be given orally. Multiple pre-clinical studies looked promising showing decreased neuronal loss and NFT which eventually led to phase 1 human trials in healthy adults which showed no significant side effects [196–200]. However, phase 2 study of ceperognastat (OGA inhibitor) in early symptomatic AD showed that those on active treatment declined more rapidly on almost all cognitive scales compared to placebo, and both dose groups had more serious adverse events. Interestingly, the treated groups lost less hippocampal volume compared to placebo, and tau PET showed statistically significant slowing of tau accumulation highlighting discrepancy between biomarkers and clinical response. Subsequently, it was announced in 2025 that Lilly had ended its development [197].

Hydromethylthionine mesylate is a tau aggregation inhibitor derived from methylene blue and it was shown to reduce tau pathology and behavioural impairment in spatial problem-solving in tau transgenic mouse models [201, 202]. It inhibits tau aggregation [203] and disaggregates pathological tau oligomers and filaments in pre-clinical models [133, 204].

Phase 3 trials in humans showed no safety concerns [205, 206] and although the initial results were reported to show no overall differences in the clinical or neuroimaging outcomes, it was later suggested that the lack of clinical difference was due to combination activity of hydromethylthionine in the majority of patients receiving 8 mg/day (low dose cohort) dose combined with lack of additional activity at higher doses. Post-hoc analysis of MCI group showed slowing of progression from CDR 0.5 to 1 in the treatment group [202, 207, 208]. Drug development is still ongoing despite primary outcomes of three phase 3 studies showing a lack of efficacy [209].

### Amyloid therapies indirectly targeting tau

Anti-amyloid therapies such as lecanemab, aducanumab and donanemab demonstrated that reducing amyloid pathology leads to the reduction of tau biomarkers in plasma and CSF [210–212]. However, the results on tau tangle pathology as measured by PET were inconsistent - for example, the donanemab phase 3 trial showed no difference in tau PET between the treatment and placebo groups, where lecanemab demonstrates a slowing of tau PET accumulation in phase 3 [213, 214]. Whether differences in outcomes between soluble tau biomarkers and tau PET is a consequence of the stage of disease or because the two pathologies are independent remains to be determined.

## In vivo biomarkers for tau pathophysiology

As AD therapeutic development advances, tau remains a central component of both its underlying pathophysiology and emerging treatment strategies, it is critically important that the field has access to tau biomarkers that capture and quantitate target engagement/therapeutic response and safety [215]. We critically review which tau biomarkers are accessible for clinical research. We consider what forms of tau they measure and whether we really have access to the cellular and subcellular compartments where tau pathology forms. Finally, we consider timing – in terms of disease stage and sampling strategy. We consider how biomarkers can help with patient selection (by staging the disease), as well as how they can serve in assessing the target engagement of tau therapies (Fig. 1).

## Neuroimaging-based biomarkers

Tau PET is the first non-invasive in vivo biomarker which directly assesses tau spatiotemporal spread in the brain – the closest alternative to reference post-mortem examination. Tau PET predicts group level changes in cognition and spatial cortical atrophy patterns over time across the clinical AD spectrum [1–3] in contrast to amyloid PET which does not correlate well with the cognitive deficit [3, 147, 216–220]. It is also able to discriminate AD from other neurodegenerative diseases [221].

Early tau radioligands used in PET (such as the hitherto only FDA- and EMA-approved [^18^F]flortaucipir (FTP), previously also known as AV1451 or T807) were shown to be elevated in patients with AD compared to controls [147, 222–225]. They bind to mixed 3R and 4R tau isoforms in PHF with higher affinity (compared to binding to 3R (pathology seen in Pick’s Disease and Frontotemporal dementia with parkinsonism-17) or 4R (pathology seen in Corticobasal Degeneration and PSP), which makes them a good biomarker candidate for AD-related tauopathy [226–228]. Other recent radioligands such as [^18^F]PI2620 have also demonstrated affinity for 4R which makes them potentially useful in 4R tauopathies mentioned above [229, 230].

Early tau PET studies attempted to recreate the spatiotemporal Braak staging of tau NFT pathology in vivo [231, 232], however, research over the past decade has highlighted the diversity of tau PET patterns as carrying diagnostic potential. Quantification of tau burden approaches based on the extraction of tau PET signal of predefined anatomical brain regions might miss meaningful signal which is why one of the largest tau PET studies thus far employed the FDA-approved visual tau PET scan assessment to establish that NFT pathology occurs in nearly 10% of cognitively normal people, 45% of people with mild cognitive impairment (MCI), and 87% of those with dementia [233]. The study also reported that for those with normal cognition, an abnormal tau PET scan signalled a nearly 60% risk of developing MCI or dementia within the next five years. Traditionally the concept of topographic spreading of tau has been prioritised over the importance of tau burden assessment, even though lessons learned from imaging-neuropathology correlation studies indicate that the former reflects a rather incomplete picture of disease [234]. These studies also demonstrated that there likely is a temporal gap between actual tau aggregation in the brain and the in vivo tau PET signal [235].

While all radioligands mentioned above bind strongly to intracellular insoluble mature tangles making them good tools for accurately assessing tau load [226, 228, 236, 237], they all show different patterns of so-called off-target binding to non-tau targets (e.g., iron, neuromelanin and monoamine oxidase B and others) [222, 238]. However, newer generation ligands (such as [^18^F]PI-2620, [^18^F]GTP1, [^18^F]RO948, [^18^F]-MK-6240, etc.) show promise with regards to greater sensitivity or less inference from off-target binding [222, 239–242]. Building on large-scale head-to-head comparisons of tau ligands to create a harmonised framework models such as CenTauR allows conversion of tracer-specific values onto a common, tracer-independent metric system [243, 244]. This is already being implemented into automated pipelines such as petBrain [245].

Tau PET remains the only modality which directly assesses the topography of tau accumulation in vivo which is extremely useful in trials and is already used in multiple studies to monitor response in terms of tau reduction [18, 150, 153].

Potential limitations of PET as a tau biomarker modality include its limited ability to detect soluble tau species that are not yet incorporated into pathological inclusions. It may not be capable of detecting successful depletion of toxic tau outside of tangles. Its sensitivity to accurately detect early successful target engagement in clinical trials remains unresolved. Practical limitations include poor access in clinical routine diagnostics, relatively high costs and the need of ligand production facilities in a vicinity that allows transport of ligands considering their half-life (20–120 min). These challenges show the importance of fluid biomarker development.

## Fluid biomarkers

Tau imaging has provided important spatial resolution, providing invaluable information about where and how much tau is present in the brain. Similar to PET findings – tau is also an excellent dynamic fluid biomarker [246–248] which correlates well with cognitive decline and AD progression, therefore is a biomarker of great interest for both diagnostic and monitoring purposes [249–253].

We critically appraise what additional information fluid biomarkers provide, and their potential practical advantages in later phase clinical trials and clinical practice. In particular, we focus on CSF and plasma tau’s ability to characterise tau and to monitor tau kinetics in vivo. The advantage of fluid biomarker over radiological biomarker is that it can measure isotopes that are potentially more specific for the clinical purpose (i.e. diagnosis, response to therapy and/or prognosis) and it should become positive earlier than radiological biomarkers and once in pipeline should be less expensive [254, 255].

**Fig. 2:**
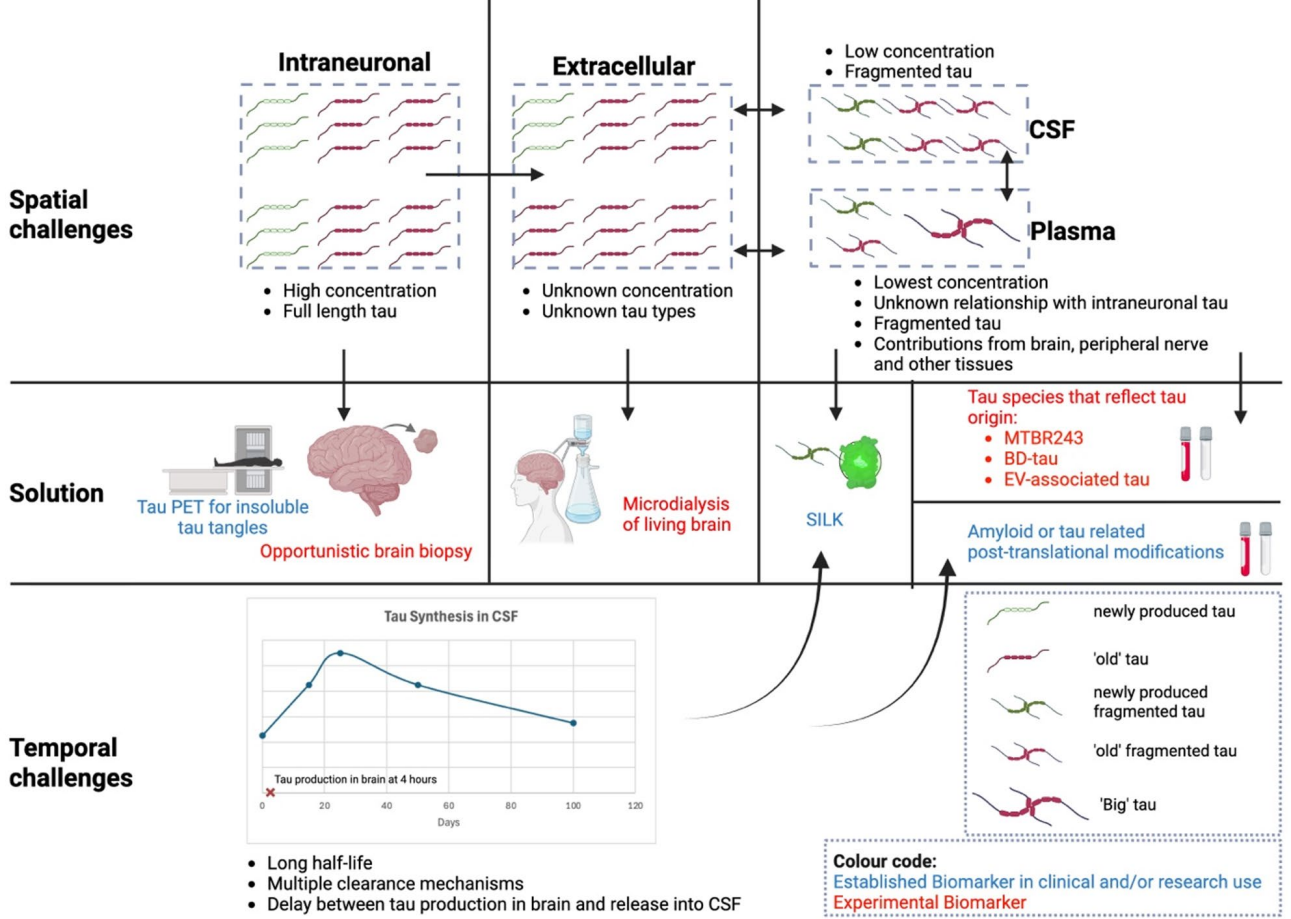
Tau biomarker spatial and temporal challenges and proposed solutions. The figure outlines how biomarkers vary in their ability to reflect tau pathology across various compartments (spatial challenge) and disease stage (temporal challenge) and proposed approaches to address the challenges (created with Biorender.com). Abbreviations: BD-tau, brain-derived tau; CSF, cerebrospinal fluid; EV, extracellular vesicle; MTBR, microtubule-binding region; PET, positron emission tomography; p-tau, phosphorylated tau; SILK, Stable Isotope Labelling Kinetics

Tau is an intracellular protein, but it can be measured in the extracellular space, CSF and plasma [256, 257]. As it undergoes truncation and phosphorylation, different species are found in different physiological compartments which provides both challenges and opportunities (Fig. 2). The mechanism by which tau exits into the extracellular space remains unclear [258]. The methods of tau release could include vesicle and non-vesicle mediated pathways, lysosomal and tunnelling nanotubes [259]. The mechanism of tau release into plasma is even more complex. There are likely breakdown pathways including filtration via blood-brain barrier, breakdown by liver and kidneys, binding to other proteins which explain the significantly low concentrations of tau. Furthermore, we know that there is also peripherally produced tau which are much bigger (110 kDa) earning the name of ‘Big’ tau compared to brain tau which is 45-65 kDa making its interpretation as a biomarker more challenging [260–263].

Total tau which is actually a mid-domain tau epitope targeted by various antibodies in clinical practice [264–266], was used separately as a ‘Neurodegeneration’ biomarker in AD Amyloid/Tau/Neurodegeneration (ATN) classification [267]. There is some evidence to suggest that elevation in CSF tau is due to axonal loss (i.e. neurodegeneration) and neuronal death leading to the release of the intracellular protein [268–270]. Studies so far show that it is not a marker of disease clinical severity [271, 272] however it does seem to reflect the disease intensity [273]. The lack of specificity meant it had limited value in distinguishing neurodegenerative diseases from one another except for cases of catastrophic neuronal loss such as Creutzfeldt-Jakob Disease (CJD) [274]. Interestingly, CSF tau levels do not increase in other forms of tauopathy [268, 275, 276] which suggests that cell death is not the only mechanism responsible for CSF tau elevation in AD and tau is not just a marker of general neurodegeneration [277]. Furthermore, studies suggest that tau can be secreted into the extracellular space from neurons independently from cell death [257, 278, 279]. This discrepancy could be simply because we have not identified other species of tau causing primary tauopathies or perhaps the rise in tau is not primarily due to tau pathology but secondary to amyloid pathology in AD.

The field of fluid biomarkers has moved forward significantly over the past decade, owing to the development of highly sensitive immunoassays and highly specific targeted mass spectrometry assays which have revolutionised how we quantitate and characterise tau in human biofluids.

Currently, CSF p-tau markers (mainly p-tau181 and p-tau217) together with Aβ 42/40 ratio are clinically accepted to be gold standard fluid biomarkers that support the diagnosis of AD with previous studies showing p-tau217 being able to distinguish patients with AD from patients with other forms of dementia and healthy controls, and being generally a more superior marker for diagnostic purposes determined by correlation with cognitive performance, brain MRI and tau PET [280–284]. However, various other phosphosites also cluster together - relevant hypophosphorylation sites (which have been shown to inversely correlate with tau and amyloid PET scans) and hyperphosphorylation sites can be used together to stage the progression of AD [285].

Other studies showed CSF p-tau231 to be a more specific marker in AD correlating with post-mortem tau pathology [286]. Interestingly, studies showed that some p-tau levels correlate better with amyloid PET which led to suggestion that secretion of p-tau217, p-tau181 and p-tau231 may represent an early reaction to amyloid plaques. In contrast other tau analytes such as MTBR-tau243 become abnormal later in disease and correlate better with tau PET and cognition [59] This raised the question on whether p-tau levels are actually part of ‘Amyloid’ or ‘Tau’ on ATN classification [267, 287, 288], which led to splitting T category into two subcategories on ATN classifications: T1 as soluble tau fragment analyte which reacts to amyloid plaques or soluble Aβ species and T2 which signals the presence of AD tau aggregates [59, 274].

Tau seed amplification assays (SAA) are also showing utility for early tau aggregation detection and discrimination between AD and other forms of dementia [289–293]. There are also efforts in using SAA as a monitoring and target engagement marker to show tau aggregation inhibition in drug trials [294].

Recent years have seen various studies showing that plasma tau markers could be as accurate as CSF in diagnostics with multiple studies showing that plasma p-tau is able to discriminate between AD and other neurodegenerative diseases [295, 296]. Multiple studies showed that plasma tau tests are similar or even superior to their CSF counterparts [297–299]. Specifically, plasma p-tau181 was shown to correlate well with amyloid and tau PET scans [246, 300, 301], however some recent data showed that plasma p-tau217 is an earlier and stronger marker associated with AD pathology having strong association with both plaques and tangles [44, 287, 300, 302–304]. Furthermore, p-tau217 and Aβ42/40 ratio showed improvement in quantitating AD pathology [287] and there is some evidence to suggest that plasma p-tau217 levels change with early amyloid accumulation before widespread tau aggregation and therefore before tau PET becomes positive making it even an earlier AD marker than tau PET [255]. Plasma MTBR-tau243 is a recent biomarker breakthrough which was shown to be an accurate diagnostic and monitoring tool known to be strongly associated with AD tau tangle pathology and AD symptoms [58]. It has an endogenous peptide form (amino acids 243–256) compared to its CSF tryptic-digest counterpart which has a tryptic peptide form (amino acids 243–254) which gives a slightly different cross-sectional profile [57, 58].

Most health systems will likely struggle to cope with expected workload of administering disease modifying therapies to patients with AD when they become widely available. Blood biomarkers can potentially help detect AD changes much earlier than imaging modalities which can make the process of screening, diagnosing and identifying the appropriate patients in the AD continuum much faster and potentially helping prioritise those patients who need to be seen sooner. Blood biomarkers also minimise the use of human resources (compared to other common investigations such as CSF sampling, MRI or PET scans) [305]. In support of this, simulations have shown that using blood biomarkers can also be very cost effective [306].

The obvious disadvantage of plasma biomarkers to imaging modalities is that they do not provide spatial information on the disease and may be confounded by various factors such as medical comorbidities, between-assay variability and sampling methods that can affect the results [307–310]. Also, there is evidence that tau protein is expressed in other peripheral tissues such as renal podocytes and muscle cells with uncertain significance to how much they affect the plasma tau results [311, 312]. However, combining various fluid biomarkers and creating a ‘staging model’ mitigates this problem reasonably well [307–310]. Furthermore, immunoassays designed to selectively measure brain-derived tau (BD-tau) in plasma by targeting tau isoforms that originate from the brain have shown promising results in differentiating AD from non-AD dementia and unaffected controls [313]. Another paper by the same group showed that BD-tau reflects the combination of pathological amyloid and neurodegeneration which is detrimental to cognitive decline in AD [288]. They also suggested that plasma BD-tau can act as a marker of neurodegeneration and paired together with plasma p-tau (which acts as a marker of amyloid) they can function as a tool to screen amyloid positive population that should be prioritised for anti-amyloid therapies [288].

Finally, the field is yet to determine whether blood biomarkers have utility and sufficient sensitivity in monitoring response to successful tau modifying therapies; incorporating plasma biomarker discovery into clinical trial design will be important for addressing this problem. Plasma tau biomarkers tend to increase after anti-tau antibody administration [314] which makes it challenging to confirm central target engagement or decide on dose selection and therefore CSF biomarkers are still needed.

However, CSF biomarkers are also not ideal. For example, the individual turnover of the proteome in CSF is likely to be dictated by a number of individual factors such as CSF production rate, clearance rate and the delivery of the drug itself may result in altered CSF dynamic. Other factors may play a part: for example, therapies such as ASO have been reported to cause ventricular volume changes which may be due to changes in CSF production/clearance discrepancy [19]. Blood-brain/CSF barrier integrity is also likely to affect the measurement of biomarkers that reflect therapeutic response to tau therapies.

Gaining confidence on how reliable CSF biomarkers are for monitoring therapy response is critical for interpreting their use in clinical trials. Measuring *rates* of production/clearance using SILK technique may partially address the challenges of using CSF biomarkers. SILK uses non-radioactive isotopes (normally ^13^C_6_) that are found in very low natural abundance and so can be used in human studies to label and track protein translation and movement around or from the CNS. By calculating the ratio of the infused isotopes and natural isotopes in tryptic peptides unique to proteins of interest, we can infer the production and clearance rates of these proteins including amyloid, tau, NfL and others [130, 143, 315]. This is especially important in drug trials as it has already been shown in mice that the absolute biomarker concentrations can be misleading in therapeutic interventions which could lead to overtreating and complications [144]. For example, in humans, tau is synthesised within neurons almost immediately [316] and released into the extracellular space in a controlled manner on day 3 after translation [130]. Since the half-life of tau in the CSF is long (around 23 days), the pool of CSF tau may not be a good representation of the large pool of newly synthesized intracellular tau. Although labour intensive, SILK is likely to be a valuable tool for early-phase clinical trials for quickly and accurately quantitating the effect on tau metabolism and ensuring that therapies are not dosed to the point of complete tau knockdown, which is likely to cause complications. This approach is currently being used in an early phase clinical trial of a tau ASO therapy where target engagement can be demonstrated acutely enabling faster decisions on dosing which can be excellent in ASO and siRNA drug classes [317]. In immunotherapy it is important to develop target engagement tests and IP-MS are especially helpful by offering more specific ways to detect specific peptide profiles targeted by the drug [57].

## Knowledge gaps in tau biomarkers and trial design

Having considered the current mechanistic approaches to targeting tau in humans and reviewing the available therapeutic strategies, we appraise the current biomarker tools, highlight gaps and propose potential solutions. As illustrated in Table 1, it is clear that for therapeutic strategies to work effectively, they need to be backed up by reliable screening, diagnostic, staging, prognostic, target engagement and/or monitoring biomarkers. While certain biomarkers are suitable for specific applications, others remain insufficient or absent.

The field also faces significant hurdles in trial design of tau therapies. Adequately powering trials is challenging when the sensitivity of available biomarker tools to detect biological change is not known, nor is the effect size we are expecting to measure. Furthermore, linking the biomarker changes to the clinical response is also a major challenge. Another major question is whether tau trials should focus on monotherapy with a tau therapy, or whether dual therapy with amyloid targeting drug will provide synergy. The timing of amyloid therapy co-administration is also an important consideration. SILK data suggesting that tau translation and secretion into CSF is increased by 200% in the presence of amyloid pathology would on one hand imply that addressing the effects of amyloid pathophysiology before targeting tau may be valuable, however on the other hand, leaving tau accumulation beyond very early stages while reducing amyloid may cause preventable damage therefore simultaneous amyloid and tau therapy may be beneficial [318].

## Conclusion

Treatments targeting amyloid pathology in AD have demonstrated the ability to slow progression in early AD. However, evidence suggests heterogeneity in response [212, 319] and due to multiple factors and pathways involved in the pathogenesis of AD we are very likely to need multiple therapies for the ‘right time for the right drug’ approach to treatment [318, 320]. Presymptomatic patients are theoretically more likely to benefit from Aβ therapy [321], but tau therapies have a chance of altering disease course, with a substantial effect size, in the symptomatic stages of the disease [130].

Drawing parallels to other disciplines where therapeutic options are more advanced such as oncology and haematology, we are very likely to eventually need a multi-therapy approach simultaneously covering multiple targets. Etalanetug is the first tau-targeted drug that is being used in combination therapy with lecanemab in a phase 2/3 study in DIAD with tau PET outcome as primary endpoint and cognitive, radiological and fluid biomarker changes as secondary endpoints [16, 212, 322, 323].

The coming decade will see a range of therapies targeting tau translation and spread through various mechanisms including the use of ASO, siRNA, active and passive immunization. Overcoming the blood-brain barrier and facilitating peripheral delivery of tau therapies to the CNS without causing off-target unwanted peripheral side effects will be one of the major logistical challenges. However, there are already some significant advances using tau ASO and transport vesicles for delivery of the drug in pre-clinical models [324]. Well planned early phase trials of tau therapies that permit the development of sensitive, accessible biomarkers of target engagement of tau therapies, which are capable of differentiating regression of tau pathological inclusions and tau spread are still needed.

Safety concerns in tau-targeting therapies so far remain low. Careful long-term follow-up of clinical trial subjects undergoing tau modification will be needed owing to its physiological role and long half-life in the CNS. Tau is also expressed peripherally in muscles and kidneys, which may bring unwanted complications [311, 312]. Biomarkers that accurately quantify the amount of tau reduction in the intracellular space, such as the use of SILK, will be important for dose determination and avoiding these unwanted safety signals [137, 325].

Unlocking success in targeting tau biology should be a strategic priority for the entire field of neurodegeneration for academic and pharmaceutical stakeholders. Multi-national cross-disciplinary efforts will be key for accelerated success and patient benefit.

**Table 1:** Gaps in AD biomarkers and proposed solutions

Problems interrogating tau biology in human clinical trials in AD	Issues	Proposed solutions	Rationale/ potential utility	Relevance to drug class
**Tempo related**				
Quantitation of *rate* of tau translation	- Quantitation of tau knockdown (safety/pharmacokinetics) - Hypersecretion of neuronal tau in AD	- Direct tau mRNA quantification - SILK - Identification of tau PTM that reflects protein age	- Quantitate tau expression at transcriptomal level where the problem arises - Quantitate tau synthesis Label-free method to quantify tau synthesis	*- MAPT* tau ASO - siRNA
Quantitation of *rate* of tau clearance	- Individual / disease specific variability in tau clearance - Clearance may be compromised by IT administration	- SILK - Autophagy biomarkers	Quantitate tau knockdown	*- MAPT* tau ASO - siRNA - Immunotherapy
**Compartment related**				
No direct access to intracellular tau pool	- Mismatch between intracellular and extracellular tau - Unknown relationship between existing fluid biomarkers and brain tau in vivo	- Sample brain, brain interstitial fluid with matched CSF and plasma ex vivo - Develop exosome-based biomarker	- Compare biomarkers in various compartments - Exosomes get released from cells and can travel to plasma which then can be measured	- PTM - Immunotherapy
Contribution of tau biomarkers from peripheral and central sources	Plasma tau has contributions from peripheral and central tau	- Specific fragmentation and phosphorylation profiles (e.g. p-tau subtypes, ubiquitination markers) - ‘Big’ tau	- Profiles that are known to be derived centrally - Expressed by peripheral and not cortical neurons	- PTM - Immunotherapy
**Spread related**				
Lack of clinical tools for capturing spread via extracellular vesicles	Tau spread by aggregation	Exosomal surface markers	Selective isolation of tau carrying vesicles	- Aggregation inhibitors - Immunotherapy
Lack of clinical tools for capturing spread via synaptic transmission	Prion-like tau spread	In vivo imaging paired with synaptic markers (e.g. SV2A)	Shows tau spread along synaptic pathways	- Aggregation inhibitors - Immunotherapy
**Structure related**				
Existing assays may miss PTM	Lack of biomarkers that easily identify various PTM	Comprehensive ‘signature’ marker reflecting the specific PTM	Tracks how modification changes with ongoing therapy	- PTM - Immunotherapy
Existing immunoassays may miss tau aggregation	Lack of biomarkers that easily identify levels of tau aggregation	Biomarker differentiating between monomeric, oligomeric and fibrillary tau species	Oligomeric tau are considered toxic prior to becoming fibrillary tau species therefore differentiating these would be useful	- Aggregation inhibitors - Immunotherapy
**Downstream biology**				
Paucity of biomarkers for capturing downstream effects of tau modification	Tau knockdown	Microtubule-associated proteins	Measures the protein responsible for microtubule stability	- Microtubule stabilisers - PTM - Immunotherapy
**Pathological specificity**				
Lack of direct markers of tau tangle pathology	Lack of biomarkers to differentiate soluble tau from insoluble tau inclusions	MTBR-tau243	MTBR-tau243 is specifically enriched in insoluble tau	- Aggregation inhibitors - Immunotherapy

## Data Availability

No datasets were generated or analysed during the current study.

## Abbreviations

Aβ: Beta amyloid
AD: Alzheimer’s Disease
ADAS-Cog: Alzheimer’s Disease Assessment Scale-Cognitive Subscale
APP: Amyloid precursor protein
ARIA: Amyloid-Related Imaging Abnormalities
ASO: Antisense oligonucleotide
ATN: Amyloid/Tau/Neurodegeneration
BD-tau: Brain-derived tau
CDR: Clinical dementia rating
CJD: Creutzfeldt-Jakob Disease
CNS: Central nervous system
CSF: Cerebrospinal fluid
DIAD: Dominantly Inherited Alzheimer’s Disease
EV: Extracellular vesicle
IT: Intrathecal
MAPT: Microtubule-associated protein tau
MCI: Mild cognitive impairment
mRNA: Messenger RNA
MRI: Magnetic resonance imaging
MTBR: Microtubule binding region
NfL: Neurofilament light
NFT: Neurofibrillary tangles
p-tau: Phosphorylated tau
PACC-5: Pre-clinical AD Cognitive Composite 5
PET: Positron Emission Tomography
PHF: Paired helical filaments
PSP: Progressive Supranuclear Palsy
PTM: Post-translational modification
SILK: Stable Isotope Labelling Kinetics
siRNA: Small interfering RNA
